# Supplementary material for: Regional Conservation and Transcriptional Regulation of Tumor-Associated Genes by macroH2A1 Deposition in Mammalian Cells
Source: Biomolecules. 2025 Sep 29;15(10):1386. doi: 10.3390/biom15101386 (PMC12562687; doi:10.3390/biom15101386)
Supplement: Supplementary file 1 [file biomolecules-15-01386-s001.zip › biomolecules-3819194_supplementary_FigureS1-S6-Proofed.pdf]

Supplementary Figures:

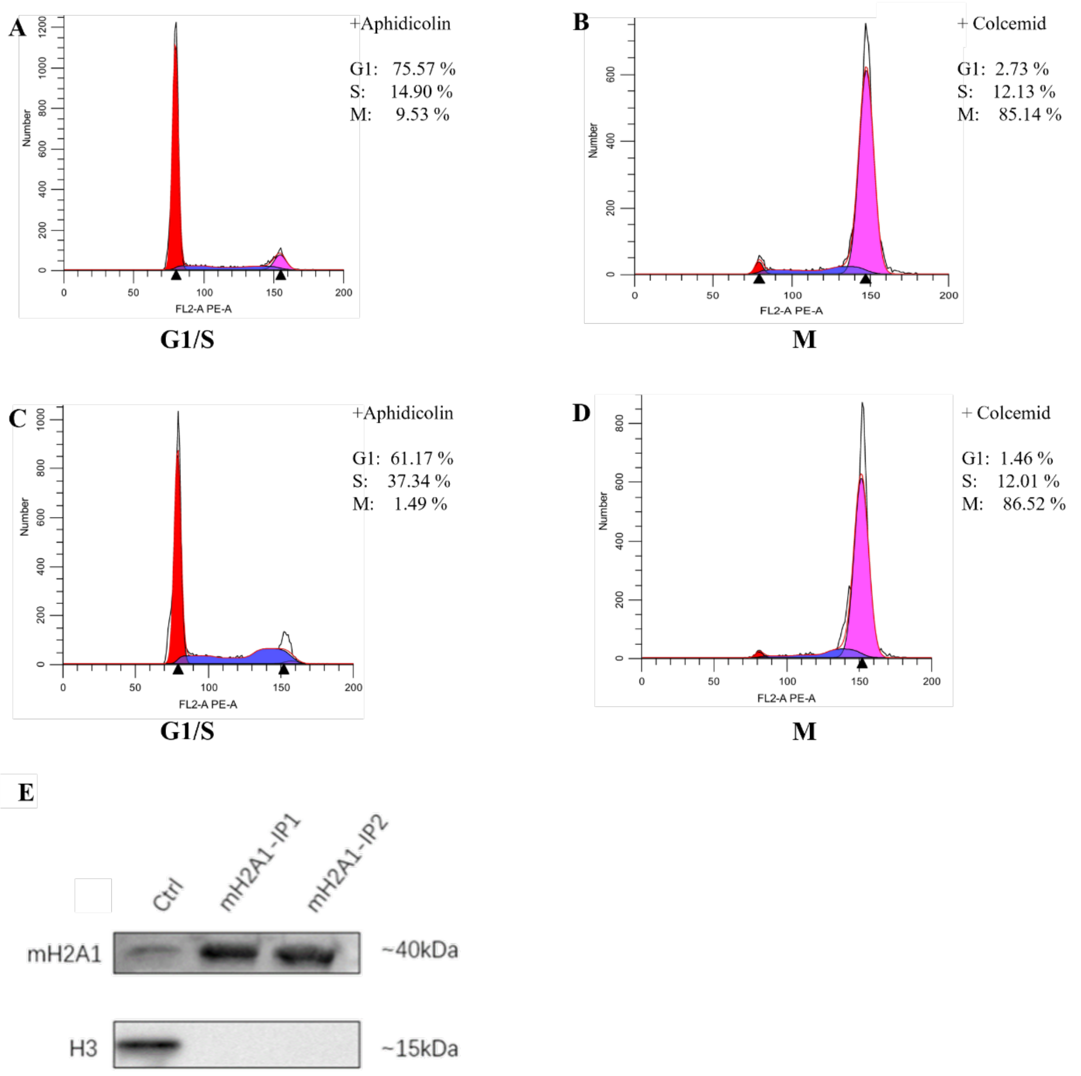

Figure S1. Results of synchronization and antibody specificity validation

(**A**) and (**B**) were flow cytometry analysis of the proportion of G1/S and mitotic cells in ARPE-19. (**C**) and (**D**) were flow cytometry analysis of the proportion of G1/S and mitotic cells in HeLa. Legends indicated the percent of cells in inset of (**A**)–(**D**). (**E**) The result of mH2A1-immunoprecipitation-western blot. The experimental groups with anti-mH2A1 antibody showed markedly stronger mH2A1 band compared with the control. Histone H3 control band was not detected, suggesting low background. These results demonstrated the ideal specificity and suitability of the antibody for subsequent ChIP experiments.

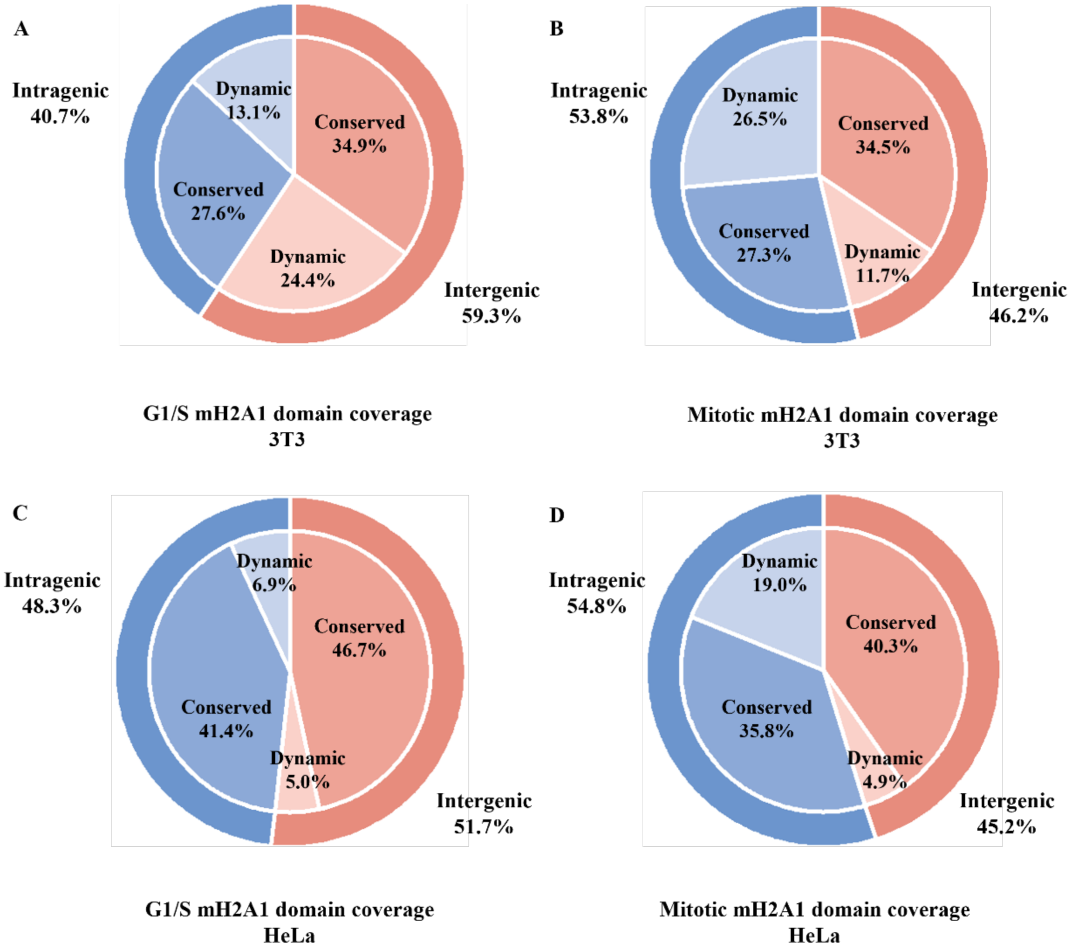

Figure S2. The genomic coverage of mH2A1 domains during cell cycle

(**A**) and (**B**) Distribution of structural and dynamic mH2A1 domains in 3T3 during G1/S and mitosis. (**C**) and (**D**) Corresponding distribution in HeLa.

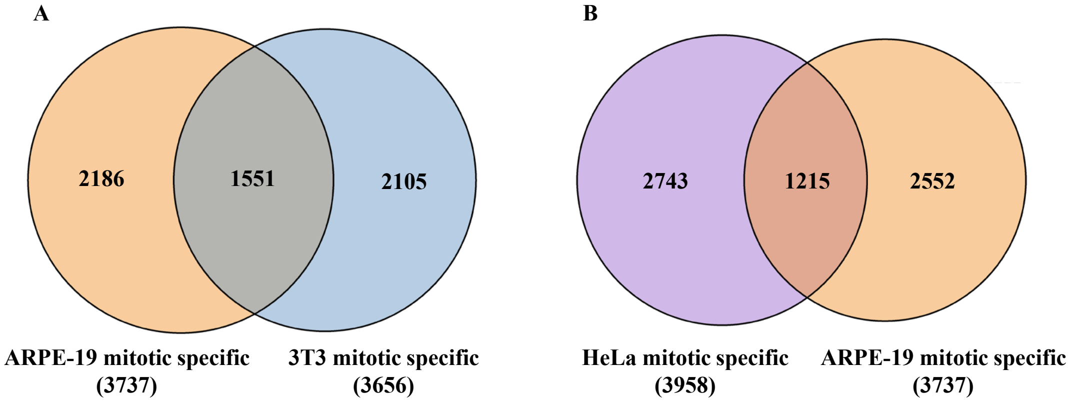

Figure S3. Comparison of mH2A1-deposition-associated genes across cell lines

(**A**) ARPE-19 vs. 3T3; (**B**) HeLa vs. ARPE-19.

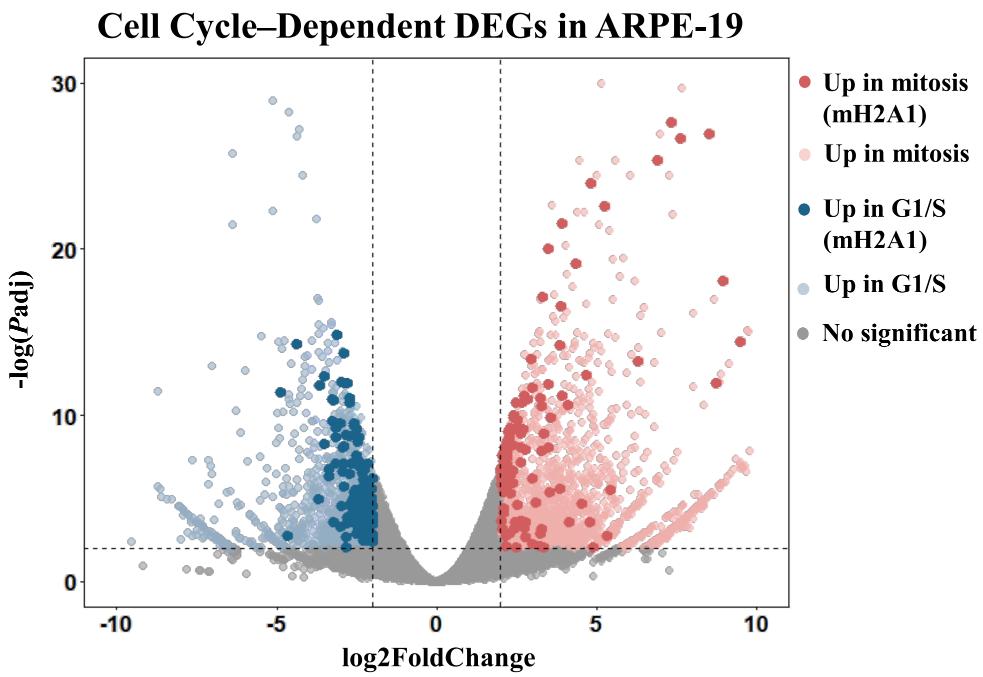

Figure S4. Cell cycle–dependent differential expression analysis of ARPE-19 cells

Genes upregulated in G1/S are shown in light navy, with mH2A1-deposition-associated genes highlighted in navy. Genes upregulated in mitosis are shown in light carmine, with mH2A1-deposition-associated genes highlighted in carmine. No significant genes are shown in gray.

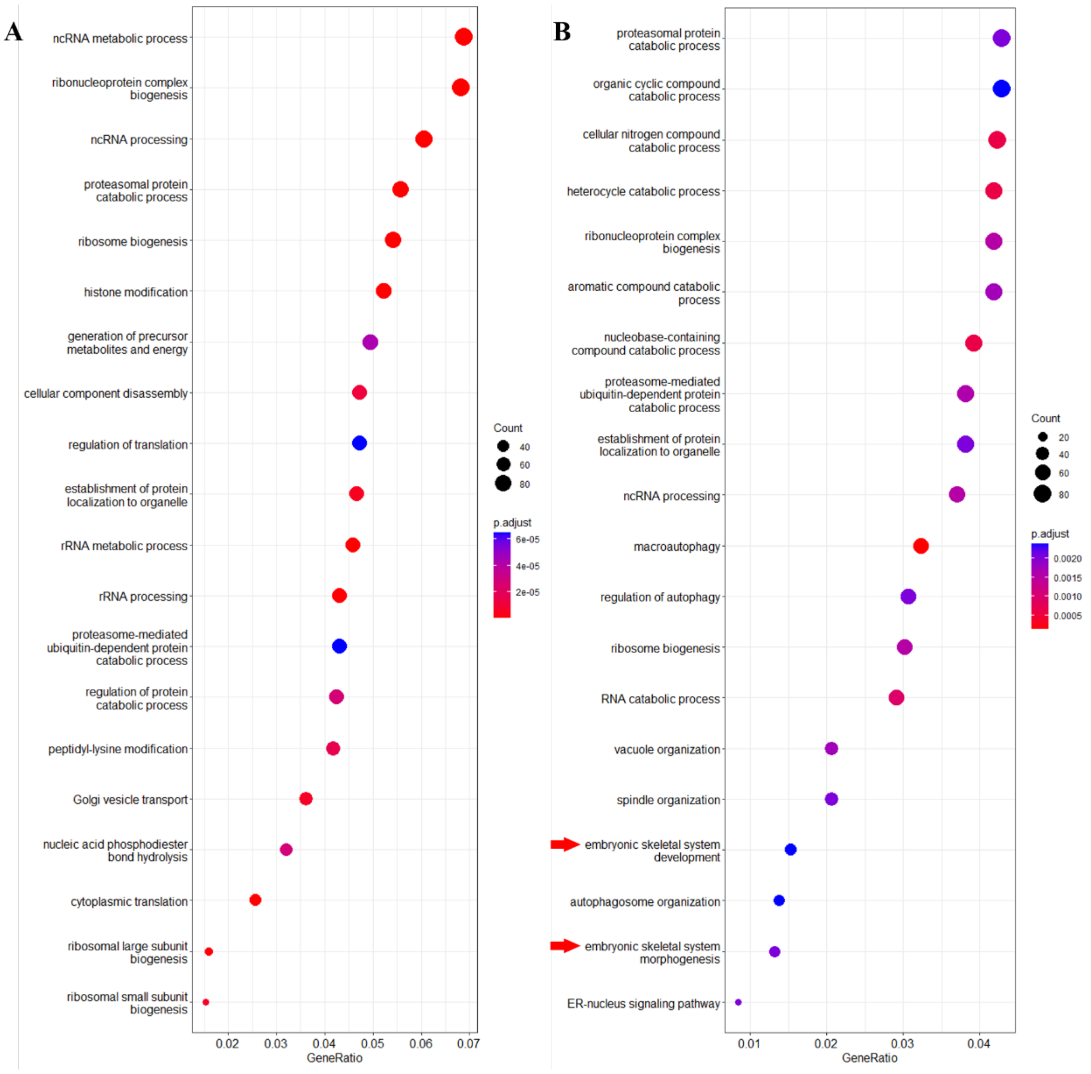

Figure S5. Functional enrichment analysis of genes covered by mH2A1-deposition-associated in 3T3 and ARPE-19 cells

(**A**) Enrichment of shared genes; (**B**) Enrichment of genes uniquely covered in 3T3. Those red arrowhead marker items are commonly related with embryonic development.

A

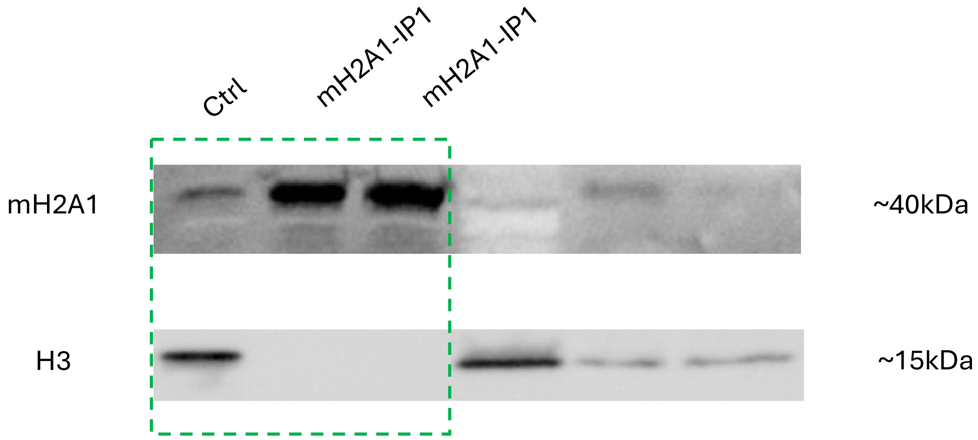

B

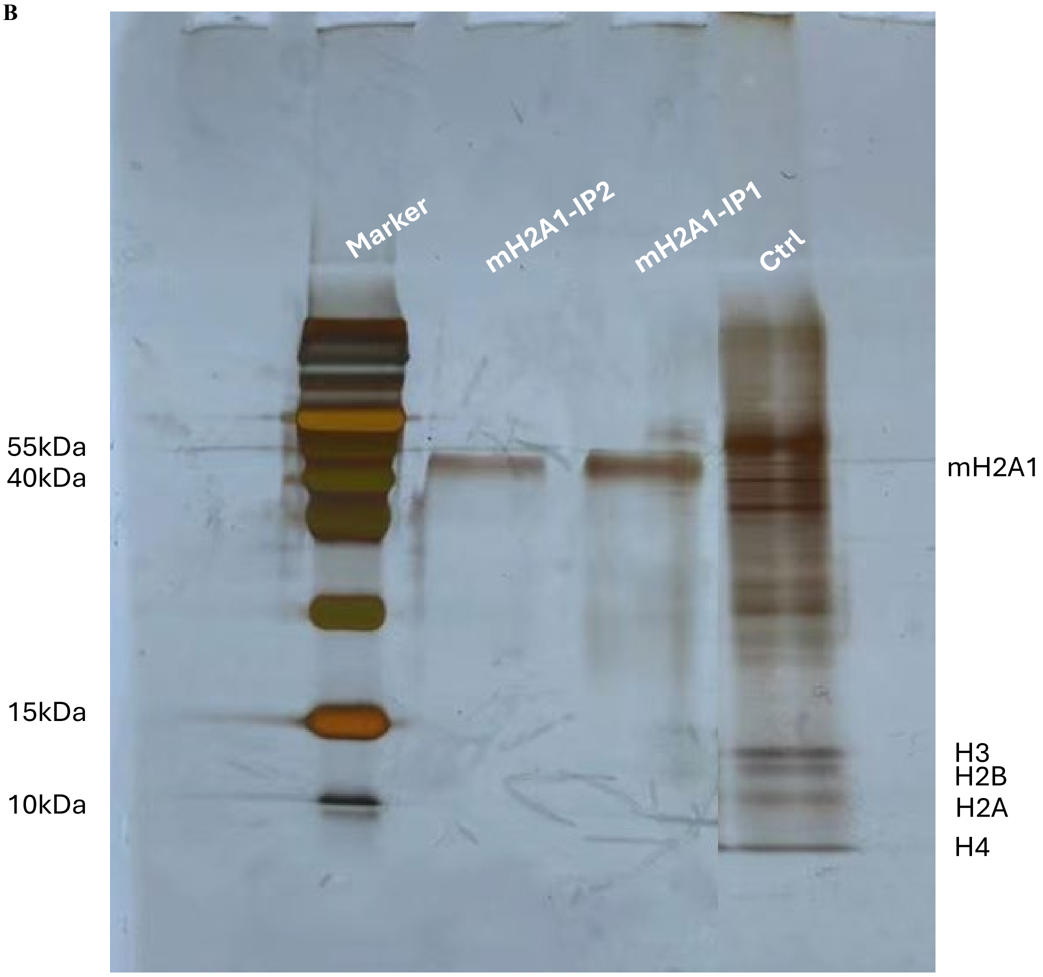

Figure S6. Original blot and gel images.

(**A**) Original blot images for Figure S1E. Proteins are transferred onto two separated nitrocellulose membranes for mH2A1 and H3 detection. After antibody incubation and exposure, lanes within the green dashed box correspond to Figure S1E. (**B**) Silver staining of the remaining portions of the above samples, as a parallel experiment, showing mH2A1 antibody specificity. Lanes (from left to right): marker, mH2A1-IP2, mH2A1-IP1, control.
